# Not Like the Textbook: An Atypical Case of Ectopic Pregnancy

**DOI:** 10.7759/cureus.29881

**Published:** 2022-10-03

**Authors:** Eleanor M Birch, Marcos Torres Molina, Joshua J Oliver

**Affiliations:** 1 Department of Emergency Medicine, Madigan Army Medical Center, Joint Base Lewis-McChord, USA

**Keywords:** anchoring bias, premature closure, cognitive bias, ectopic pregnancy, emergency medicine

## Abstract

Ectopic pregnancy is a potentially life-threatening outcome of pregnancy that occurs with the implantation of an embryo outside of the endometrial cavity. Classically considered a “must not miss” diagnosis, ectopic pregnancy is a common emergency department presentation, associated with a symptom triad of amenorrhea, vaginal bleeding, and abdominal pain. However, varied presentations of ectopic pregnancy or lack of typical risk factors can complicate the evaluation and diagnosis of this condition. This case report describes an atypical presentation of ectopic pregnancy after a reported spontaneous abortion, in which the patient was initially discharged with a diagnosis of pelvic inflammatory disease. This case provides an illustration of ectopic pregnancy that presented without classically associated symptoms, and also highlights how anchoring bias and pre-emptive closure, among other cognitive biases, contributed to a missed diagnosis.

## Introduction

Ectopic pregnancy, defined as a pregnancy occurring outside of the uterine cavity, is a common emergency department diagnosis, occurring in 12.3 out of every 1,000 live births [1]. Ectopic pregnancy remains a leading cause of death in early pregnancy, and delayed diagnosis is associated with significant morbidity and mortality [2,3]. In this report, the authors present an atypical case of ectopic pregnancy. We aim to highlight both the varied clinical presentations of ectopic pregnancy and the cognitive biases that may prevent prompt diagnosis, including anchoring bias, pre-emptive closure, representativeness bias, and confirmation bias. This case was previously presented as a poster at the 2022 GSACEP conference on April 9, 2022.

## Case presentation

An otherwise healthy 25-year-old G3P1021 presented to the emergency department overnight complaining of lower abdominal pain for one day. She reported that two weeks prior to presentation she had experienced a spontaneous, uncomplicated abortion at seven weeks gestation at an outside hospital. No records from this hospital were available at the time of her presentation. She reported no history of sexually transmitted diseases or use of assistive reproductive technology. On presentation to the emergency department, she was reporting intermittent bilateral pelvic pain and pain with urination, but she reported that she had not experienced fevers, nausea, vomiting, or diarrhea. On exam, she was afebrile and mildly tachycardic with a heart rate of 110, blood pressure of 121/77, and oxygen saturation of 98% on room air. Her abdominal exam was notable for bilateral lower quadrant tenderness to palpation and a positive Murphy sign with no distention, rebound, or guarding. She had cervical motion tenderness on pelvic exam without discharge or bleeding. At this time, given the patient’s history, cervical motion tenderness, and right upper quadrant pain, this presentation was thought to represent pelvic inflammatory disease, with possible early Fitz-Hugh-Curtis syndrome.

**Figure 1:**
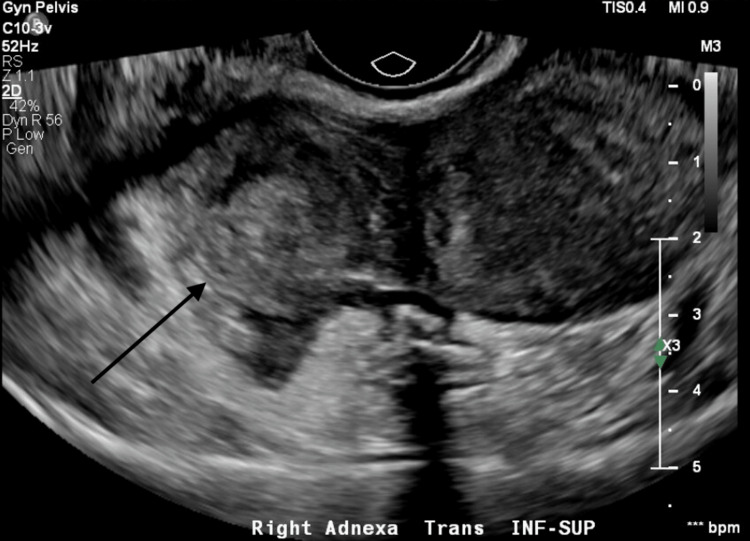
Transvaginal ultrasound of the patient's right adnexa, with heterogenous mass identified (arrow).

Labs revealed a bHCG of 308. A right upper quadrant ultrasound was unremarkable with no hepatic or biliary abnormalities, and a transvaginal ultrasound, seen in Figure 1, revealed a right-sided heterogeneous mass and moderate free fluid in the pelvis. The overnight imaging was initially read as nondiagnostic by a resident, though they reported that the findings could indicate a tubo-ovarian abscess or ectopic pregnancy. Of note, the radiology resident suggested consulting obstetrics and gynecology if there was a clinical concern for ectopic pregnancy. The patient's initial symptoms were treated with a fluid bolus and 1000mg acetaminophen with significant improvement of her abdominal pain and resolution of her tachycardia.

Obstetrics and gynecology were consulted and determined that the patient’s presentation was most consistent with pelvic inflammatory disease with a lower suspicion for tubo-ovarian abscess given her reassuring appearance and the resolution of her pain. Her elevated bHCG was thought to represent sequelae of recent spontaneous abortion. They recommended trending of bHCG every 48 hours. After a discussion with the patient, they prescribed ceftriaxone, doxycycline, and metronidazole for pelvic inflammatory disease and recommended follow-up in the clinic the next day.

The following morning, an attending radiologist reviewed the patient’s imaging and identified the ovarian mass as an ectopic pregnancy. The patient was contacted and she returned for evaluation at the obstetrics and gynecology clinic. She had remained hemodynamically stable and without a recurrence of her pain since discharge. She underwent an operative laparoscopy with right salpingectomy which revealed hemoperitoneum and an apparent ruptured ectopic pregnancy at the isthmus of the right fallopian tube. She tolerated the procedure well and was discharged on postoperative day one in stable condition.

## Discussion

Ectopic pregnancy is a high-risk condition that may present a diagnostic challenge in the emergency department. While the typical presentation of ectopic pregnancy includes a triad of amenorrhea, abdominal pain, and vaginal bleeding, these symptoms occur in only one-third of cases [4]. Because ectopic pregnancy in an unstable patient is a surgical emergency, it is imperative that emergency physicians maintain a high suspicion for ectopic pregnancy in any patient with abdominal pain who can become pregnant [5]. However, several factors including time constraints, task switching, and reliance on heuristics may increase diagnostic error in atypical cases [6].

Misdiagnosis in the emergency department is common, and it contributes to increased return visits and delays in appropriate intervention [7]. Common errors include failure to order appropriate diagnostic tests, inadequate history taking, and failure to follow up on abnormal test results [8]. The majority of missed diagnoses in the emergency department can be attributed to cognitive errors [9]. Inexperience, inadequate knowledge, and cognitive biases contribute to these cognitive errors [10]. In this case, several errors provide illustrative examples of the effects of cognitive bias in patient evaluation.

Anchoring bias, pre-emptive closure, confirmation bias, and representativeness bias were significant contributors to the delay in diagnosis in this case. Focusing on one diagnosis early in the treatment course while failing to adjust for other possibilities is anchoring bias. Occurring later in the diagnostic process, premature closure is when a provider accepts an early or initial diagnosis without searching for information that may point to another diagnosis. Interpreting new findings to confirm preexisting beliefs is confirmation bias. Finally, representativeness bias occurs when the perceived frequency of a diagnosis, or the perceived similarity of the current presentation to other recent presentations of a disease, affects a provider’s assessment of how likely a patient is to have the disease [11-13].

These cognitive errors can be seen throughout the diagnostic evaluation of this patient, from initial differential diagnosis to the response to consultants’ recommendations. Upon initial presentation, the patient’s symptoms were assumed to be related to pelvic inflammatory disease due to the physical exam findings of cervical motion tenderness; this remained the leading diagnosis throughout the evaluation, illustrating an example of anchoring bias. Later, the patient was discharged with a diagnosis of pelvic inflammatory disease despite this diagnosis inadequately explaining the ultrasound findings, which demonstrates premature closure. At that time, several diagnostic possibilities remained that had not been adequately assessed. Despite the patient’s report of recent spontaneous abortion, for which medical record confirmation was not available, ectopic pregnancy was not adequately ruled out. Spontaneous abortion had not been confirmed, and heterotopic pregnancy should have remained a consideration. Though rare, occurring in only between 1 in 4,000 to 1 in 30,000 pregnancies, heterotopic pregnancy is becoming more common, largely due to the increased use of assistive reproductive technology [14]. In addition, the elevated bHCG was interpreted to confirm the pre-existing belief that the patient had previously experienced a spontaneous abortion, rather than confirming the possibility of a current pregnancy, revealing the effect of confirmation bias. Right upper quadrant tenderness was also interpreted to indicate possible early Fitz-Hugh-Curtis syndrome, as that aligned with the belief that the patient had pelvic inflammatory disease. However, this finding can be caused by alternative diagnoses, including hemoperitoneum. Finally, representativeness bias likely contributed to the diagnostic delay in this case. Ectopic pregnancy in a population of patients with recent intrauterine pregnancy in the absence of assistive reproductive technology is rare, and so the diagnosis was considered much less likely than other more common diagnoses in this population. In addition, the patient’s exam and clinical improvement after volume resuscitation did not match the common mental model of a patient with ectopic pregnancy, despite other elements of the evaluation pointing to this diagnosis. This made the discharge diagnosis of pelvic inflammatory disease seem more acceptable, and it falsely reassured the team against the possibility of ruptured ectopic pregnancy.

## Conclusions

This case describes an atypical ectopic pregnancy presentation leading to delayed diagnosis and an illustration of the ways in which cognitive biases can negatively affect emergency department evaluations. We aim to emphasize the varied ways in which ectopic pregnancy may present, including the absence of vaginal bleeding or persistent severe abdominal pain. However, we also hope to highlight cognitive biases in order to encourage awareness of diagnostic pitfalls, even when approaching common diagnoses.

Disclaimer

The views expressed are those of the author(s) and do not reflect the official policy of the Department of the Army, the Department of Defense, or the U.S. Government. The investigators have adhered to the policies for protection of human subjects as prescribed in 45 CFR 46.

## References

[1] Mann LM, Kreisel K, Llata E, Hong J, Torrone EA (2020). Trends in ectopic pregnancy diagnoses in United States emergency departments, 2006-2013. Matern Child Health J.

[2] Creanga AA, Shapiro-Mendoza CK, Bish CL, Zane S, Berg CJ, Callaghan WM (2011). Trends in ectopic pregnancy mortality in the United States: 1980-2007. Obstet Gynecol.

[3] McGurk L, Oliver R, Odejinmi F (2019). Severe morbidity with ectopic pregnancy is associated with late presentation. J Obstet Gynaecol.

[4] Ranji GG, Usha Rani G, Varshini S (2018). Ectopic pregnancy: Risk factors, clinical presentation and management. J Obstet Gynaecol India.

[5] Robertson JJ, Long B, Koyfman A (2017). Emergency medicine myths: ectopic pregnancy evaluation, risk factors, and presentation. J Emerg Med.

[6] Fordyce J, Blank FS, Pekow P (2003). Errors in a busy emergency department. Ann Emerg Med.

[7] Halsey-Nichols M, McCoin N (2021). Abdominal pain in the emergency department: missed diagnoses. Emerg Med Clin North Am.

[8] Medford-Davis L, Park E, Shlamovitz G, Suliburk J, Meyer AN, Singh H (2016). Diagnostic errors related to acute abdominal pain in the emergency department. Emerg Med J.

[9] Kachalia A, Gandhi TK, Puopolo AL (2007). Missed and delayed diagnoses in the emergency department: a study of closed malpractice claims from 4 liability insurers. Ann Emerg Med.

[10] Ely JW, Graber ML, Croskerry P (2011). Checklists to reduce diagnostic errors. Acad Med.

[11] Kilner T, Butterfield E, Poonian J (2020). Common cognitive pitfalls in emergency medicine. Emerg Med Australas.

[12] Hansen K (2020). Cognitive bias in emergency medicine. Emerg Med Australas.

[13] Daniel M, Khandelwal S, Santen SA, Malone M, Croskerry P (2017). Cognitive debiasing strategies for the emergency department. AEM Educ Train.

[14] American College of Obstetricians and Gynecologists’ Committee on Practice Bulletins—Gynecology (2018). ACOG Practice Bulletin No. 193: tubal ectopic pregnancy. Obstet Gynecol.

